# Employee Sustainable Performance (E-SuPer): Theoretical Conceptualization, Scale Development, and Psychometric Properties

**DOI:** 10.3390/ijerph181910497

**Published:** 2021-10-06

**Authors:** Tianchang Ji, Jan de Jonge, Maria C. W. Peeters, Toon W. Taris

**Affiliations:** 1 Department of Social, Health and Organisational Psychology, Utrecht University, P.O. Box 80140, NL-3508 Utrecht, The Netherlands; t.ji@uu.nl (T.J.); m.peeters@uu.nl (M.C.W.P.); t.taris@uu.nl (T.W.T.); 2 Human Performance Management Group, Department of Industrial Engineering and Innovation Sciences, Eindhoven University of Technology, P.O. Box 513, NL-5600 Eindhoven, The Netherlands

**Keywords:** employee sustainable performance, work performance, counterproductive work behavior, employee vitality, scale development, psychometrics

## Abstract

Although the concept of employee sustainable performance has received considerable attention in the practitioner literature, academic research still lacks a clear conceptualization and empirical operationalization of this concept. Defining employee sustainable performance as a regulatory process in which an individual worker enduringly and efficiently achieves particular desired work goals while maintaining a satisfactory level of well-being, this paper describes a corresponding instrument called E-SuPer, and examines its psychometric properties. The E-SuPer instrument was tested and cross-validated using two cross-sectional survey studies (*n* = 153 and *n* = 160), focusing on factorial validity, internal consistency, and discriminant and concurrent validity. Psychometric findings across the two samples revealed that the E-SuPer instrument consists of one general factor of ten items with good internal consistency. Discriminant validity and concurrent validity with other relevant constructs (task performance, counterproductive work behavior, and employee vitality) were also confirmed, showing promising results. Finally, theoretical and practical implications, as well as suggestions for future research, are outlined.

## 1. Introduction

The past two decades have witnessed a growing interest in building sustainability for ensuring the performance of both organizations and employees in the long haul [1,2,3,4,5]. “Sustainability” and “performance” have by now become widely used terms. However, in the academic literature, these two topics are rarely studied together. This is unfortunate, as high employee performance may, in the short run as well as in the long run, have adverse consequences for employee health and well-being, which could in turn negatively affect later employee performance [6,7,8,9]. In this study, we argue that in order to promote employees’ future job performance (i.e., to make it *sustainable*), it is imperative to simultaneously consider both employee performance and employee health and well-being [10,11].

Previous research has established a conceptual assessment framework to identify what sustainability means to a specific organization and how existing programs and initiatives can be used to improve the sustainability of an organization’s overall performance [12]. Interestingly, the value of sustainable performance at the individual level, i.e., for employees rather than the organizations they work for, is often overlooked or even neglected. In our research, we explore sustainable performance from an individual perspective; that is, employee sustainable performance at work. Based on this status quo, there are still many gaps in the theoretical and empirical knowledge surrounding employee sustainable performance. Importantly, the concept of employee sustainable performance is still in an early stage of development, and appropriate conceptual models and theoretical frameworks are still to be built and tested [1]. Thus, insight into the factors related to employee sustainable performance is important in benefiting employees—not only scientifically but also from a practical point of view.

From another perspective, organizations need employees to be able to perform in a sustainable way (i.e., to perform well during a longer period of time), to improve their competitiveness. Since organizations must deal with demographic trends such as ageing, longer working lives, and a stable or even decreasing influx of youth, keeping employees working and productive “for life” is appealing and much-needed. Therefore, we argue that exploring employee sustainable performance and promoting its formation is of great significance to employee health, well-being and development, to the organizations these employees work for, as well as to building social sustainability [1,4].

Based on these viewpoints, this article aims to develop and validate an instrument that taps employee sustainable performance; that is, an optimal combination of (or balance between) employee performance and employee well-being and health, that is warranted in the long run. In pursuing this goal, this research presents the first empirical study that contributes to the development and validation of the instrument.

### 1.1. Theoretical Background

Most of what has been written about employee sustainable performance (abbreviated E-SuPer) can be found in practitioner-oriented journals or reports, where it has its basis in practice rather than in theory and empirical research [13]. There is surprisingly limited academic and empirical research on a topic that has become so popular.

In the practitioner literature, E-SuPer has mostly been used to depict an ideal working state of an employee in both the present and the future. Alluding to the Brundtland commission [14], some human resources management trainers have extended the definition of sustainable development to include sustainable employee performance; that is, “an ideal working state in which performance can meet present working needs without compromising future performance to meet future working needs” [14] (p. 43). They consider that creating E-SuPer is an important way to achieve sustainability of both employees and the organizations they work for. More and more well-known companies and organizations (such as Unilever, Huawei, and Lenovo) are integrating “sustainability” into their efforts to improve employee performance and strategies to achieve long-term goals. For that reason, E-SuPer is greatly promoted in the field of practice. However, as said before, there are still important questions waiting to be answered from an academic perspective.

Given the limited amount of empirical research on E-SuPer, so far, there has been little theory and model development in this area [1]. However, two streams of research stand out in providing some guidance for conceptualizing this concept. The first stream of research was initiated by Spreitzer and her colleagues [13,15]. When focusing on *thriving at work*, they introduced a concept similar to employee sustainable performance at work. Thriving is described as a positive psychological state including (active) learning (presumably leading to high performance) and vitality (representing a dimension of well-being). The notion of thriving plays an important role in describing the sustainable performance of employees, with empirical evidence showing that people who thrive demonstrate better overall performance (as reported by their managers) and much less (self-reported) burnout than their peers [13,15,16]. In addition, they were more committed to the organization and more satisfied with their jobs. They also missed much less work and reported fewer doctor visits, which meant health care savings and less lost time for the company [15,16]. Although high levels of performance are not explicitly at the core of the thriving concept, in terms of its emphasis on employee health and well-being, the stream of research on thriving is surely relevant to E-SuPer. That is, sustainable performance is expected to coincide with high levels of energy and low levels of burnout. Moreover, employees with high scores on sustainable performance do not only enjoy a higher level of well-being and are more productive than their peers, but they also engage in creating a common, sustainable future for organizations and themselves. They are not sprinters but marathon runners [16]. Therefore, since this approach assumes that employee well-being and performance are interrelated, it is important to make sure that employees are happy, healthy and productive. This implies that the concept of thriving encompasses the perspective of sustainability. Moreover, the sustainability notion implies a healthy balance between employee performance and well-being in the long run. In other words, the body of research on thriving supports the idea that high levels of E-SuPer are associated with beneficial outcomes for both the employee and the organization.

The second stream of research is based on the concept of *vitality at work*. Some scholars propose that employee vitality is a crucial aspect of E-SuPer [1,17,18]. In order to test some of the assumptions underlying employee vitality as a dimension of the E-SuPer concept, Dorenbosch [16] analyzed survey data from nearly 2000 Dutch employees, providing empirical support for these assumptions. Building on Conservation of Resources (COR) theory [19,20] and the proactive work behavior literature [21], Dorenbosch [17] found that three resource constraints might threaten E-SuPer over time: time constraints, energy constraints, and competence constraints. That is, employees reported higher levels of sustainable work performance in situations offering them both high resource levels and adequate resource regeneration.

Taken together, these streams of research emphasize that (1) employee well-being and performance are intertwined, (2) these concepts should be considered from a lifelong perspective, and (3) employees reported higher levels of sustainable work performance in situations offering them both high levels of resources and adequate resource regeneration.

### 1.2. Towards a Conceptualization of Employee Sustainable Performance

To access the relevant literature, the evidence presented in previous research [2,3,4,16] showed that for the construction of E-SuPer, it is crucial to get an understanding of sustainability and employee performance. Sustainability is a concept derived from ecology, referring to the capacity of systems and processes to develop, grow and care, and endure [22]. Sustainability can also be defined as an effort to conserve, use, and recycle natural resources in an efficient way [1]. Today’s discussion of sustainability has long gone beyond its original narrow scope and involves the intersection and integration of many disciplines and research fields, especially in the organizational management area. This raises an important question: How can employee performance be made sustainable?

Regarding theories on employee performance, Campbell and his associates [23] suggested that “Performance is what the organization hires one to do, and do well” (p. 35). Thus, employee work performance can be considered as the total expected value to the organization of the discrete behavioral episodes that an individual carries out over a standard period of time [24,25]. Specifically, performance refers to behavior as well as results [23,24,25,26,27,28]. However, “behavior” and “performance” are not the same. While behavior is generally considered to refer to what people do, performance is the expected organizational value of this behavior. Results are those individual behaviors that help or hinder the organization from achieving its goals, which makes it attractive to focus on results when considering individual performance [24]. This implies that good performance is an outcome that satisfies the person or the organization, and that is caused by effective employee behaviors that help an organization in achieving its objectives.

*Defining E-SuPer*. Sustainable performance in relation to work was first introduced by Docherty and colleagues [2] when discussing sustainable work systems. They argued that sustainable work systems are systems where human, job, and social resources are regenerated and renewed through the process of work while still maintaining productivity. Sustainable work systems can be considered an important key for sustainable work performance and for maintaining long-term human sustainability. Peeters and her colleagues [29] define E-SuPer directly as “maximizing work performance as well as worker health and well-being” (p. 5). Thus, in this view, E-SuPer refers to an ideal, sustainable, psychological state when occupying and performing an organizational role. Next, De Jonge and Peeters [1] argue that E-SuPer involves a crucial component in addition to work performance; that is, employee vitality. Vitality at work refers to being “energetic and strong, and [feeling] physically and mentally well” [1,29] (p. 533). Finally, Dorenbosch [17] defines E-SuPer as “a joint function of high resource levels (energy, time, and competencies) and the allocation of resources which also allows for resource regeneration” (p. 156). He further states that constraints on resources can threaten the effective allocation of these resources, which might, in turn, threaten sustainable work performance over time. Finally, to deal with constraints or demanding aspects at work, employees need access to sufficient resources [30]. This involves regulatory processes to cope with states of psychological imbalance induced by demanding aspects at work [31,32,33].

**Figure 1:**
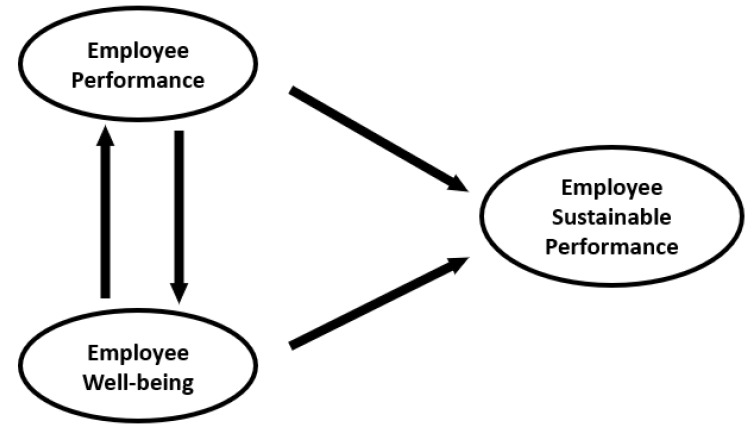
Proposed conceptual framework of the present study.

Therefore, E-SuPer comprises two main determinants: (1) employee performance and (2) employee well-being—especially vitality. We assume that both concepts may mutually influence each other and that to maximize employee performance, in the long run, it is imperative to make sure that employee well-being and performance are not threatened by an overly strong focus on either of these concepts. Rather, from a long-term perspective on performance it is important that a good balance between health and well-being is preserved as a precondition [34]. Thus, in line with the aforementioned theoretical context and former research, we define E-SuPer as a regulatory process in which an individual worker enduringly and efficiently achieves particular desired work goals while maintaining a satisfactory level of well-being. Moreover, this process involves a continuous (re)generation and preservation of resources invested while achieving these goals, and these goals usually refer to desired outcomes for the individual, as well as for this individual’s environment. Figure 1 shows the corresponding conceptual framework.

### 1.3. Present Study

In a quickly changing and dynamic work environment, E-SuPer is becoming a more appealing concept, where both the employee and the organization could benefit from. Although the importance of E-SuPer is increasing, recent academic research in this area has not yielded an instrument tapping this concept. Obviously, a clear conceptualization is a prerequisite for any valid instrument, including a measure of E-SuPer [35]. To fill the blanks in current research on E-SuPer and to bring this concept more into the spotlight, our research started from the following research question: What is employee sustainable performance, and how can it be measured? And how does it relate to other relevant constructs such as employee performance and well-being? Addressing these questions requires a valid and reliable measurement instrument for E-SuPer. To achieve this aim, this article draws on existing literature and qualitative inquiry to provide a conceptual working definition and to generate scale items. Then, a cross-validation method was applied to further validate the proposed scale, as well as associations with closely related constructs.

Empirical research was conducted to validate the new instrument on two independent samples of employees. The empirical part of this study had three additional aims: (1) to identify the underlying factor structure by exploratory factor analysis and confirmatory factor analysis; (2) to assess scale reliability; and (3) to validate the instrument using discriminant validity and concurrent validity, as well as to establish a nomological network of the new instrument. In examining the validity of E-SuPer, we will relate it to two measures of work performance (i.e., task performance and counterproductive behavior—representing the productivity dimension) and employee vitality (representing the well-being dimension). Based on our literature review, we assume that high levels of E-SuPer will relate positively to task performance (Hypothesis 1) and employee vitality (Hypothesis 2), and negatively to counterproductive work behavior (Hypothesis 3).

## 2. Materials and Methods

### 2.1. Research Design and Participants

This study was approved by the ethical board of the Faculty of Social Sciences of Utrecht University, the Netherlands (registration numbers #20-0654 and #21-0035). Two survey data sets were collected for this study: an exploratory data set (Sample A) and a confirmatory data set (Sample B). In both cases, an anonymous online survey was conducted among the potential participants. They received an email in which the purpose of the project was briefly described, after which they were invited to participate. The confidentiality and anonymity of their answers were emphasized and assured, and participation was voluntary. For those who wished to participate, a link to an electronic questionnaire (posted on Qualtrics) was attached. Participants were asked to fill out an online questionnaire about their working conditions in the previous four weeks, work characteristics, health, well-being, work/home interactions, and (sustainable) work performance. To qualify for the study, participants were required to be employed for at least 12 h per week and to be at least 18 years old. Participants were asked to provide informed consent, after which they could proceed with the main questionnaire. The final overall sample size was 313 participants: 153 people in Sample A and 160 people in Sample B.

Sample A was collected in January 2021. The participants of sample A included members of two networks of the International Commission on Occupational Health (ICOH) association. The first network was the ICOH Scientific Committee on Work Organization & Psychosocial Factors (ICOH-WOPS), and another was the ICOH Scientific Committee on Cardiology in Occupational Health (ICOH-CVD). The ICOH-WOPS is an organization that aims to promote awareness, research, and education, disseminate good practices, and influence policy development in the area of work organization and psychosocial factors. The ICOH-CVD serves a professional network of researchers, occupational health physicians, and public health workers throughout the world concerned about the impact of work and work-related factors on cardiovascular health. To enlarge the sample size, additional participants were recruited via the ICOH members, authors’ professional networks and social media. In total, 153 participants completed the survey. Due to the nature of the data collection procedure, the actual response rate for this study was unknown. Participants were on average 43.2 years old (SD = 12.8) and 65.4% was female. 53.6% held a doctoral degree and 35.3% held a master’s degree. The average working experience was 19.7 years (SD = 12.6). Among them, 39.9% of participants were from the Netherlands and 14.4% of them were from the US. The remaining participants were mainly from European countries. Finally, 66.0% of the participants were employed in the education and health services sector.

For cross-validation reasons, the lead author assigned the electronic questionnaire to three large-scale Chinese organizations (a vocational training college focused on tourism; a Chinese manufacturing company; and a public administration office, all based in Anhui, China) in April 2021. Out of these organizations, 268 participants completed the questionnaire voluntarily after being informed of the confidentiality principle. Those providing no valid responses or who failed the quality control items (i.e., “1 + 1 = 2, please click strongly agree”) were excluded from further analysis, yielding a final sample size of 160 participants (59.7% of the initial responses). Fifty-three percent of these participants were female, and the average age of this dataset was 36.3 years (SD = 10.9), their average working experience was 13.3 years (SD = 11.3). 40.6% of the participants held a bachelor degree; 13.8% held a master’s degree; and 2.5% held a doctoral degree. Sample B differed significantly from Sample A in terms of age, working years, and educational level: *t*(308) = −5.12, *p* < 0.001, *t*(308) = −4.76, *p* < 0.001, and *t*(311) = −31.72, *p* < 0.001, respectively.

### 2.2. Measures

For the purpose of this study, the items of the Sample A survey (that was provided in English) were translated into Chinese for the Sample B survey by the lead author. To ensure accuracy and clarity of expression, a double back-translation procedure was conducted and reviewed by an English background professional and a Ph.D. student in Occupational Health Psychology [36].

*Employee sustainable performance*. For the development of the present new instrument for employee sustainable performance, we conducted a literature review to search for closely related and well-validated instruments in the area of employee work performance [35,37,38]. Out of three apparently useful instruments, we chose Goodman and Svyantek’s [36] measure as the most appropriate due to its clear conceptualization and good psychometric qualities. Part of this instrument consists of nine items assessing employee’s task-based work performance (e.g., achieving the objectives of the job as well as planning and organizing the job’s objectives) and promotion expectations (e.g., appearing suitable for a higher-level role). Consequently, these nine items of the task-based work performance scale of Goodman and Svyantek [37] were adapted and molded into a new instrument for E-SuPer. Moreover, the original item ‘Plans and organizes to achieve objectives of the job and meet deadlines’ was divided into two items in which “objectives of the job” and “meet deadlines” were separated, resulting in ten items in total. As sustainable work performance is about work performance in the long run, and ideally during an employee’s whole career, we integrated this notion into this new instrument. Therefore, we included terms that referred to high performance during a more sustainable, lifelong period. The exordium of the original instrument was also changed to align with the conceptualization of E-SuPer. Specifically, the original exordium was changed from “At this moment …” to “During my entire career, I will be able to …”. Finally, we changed the original 7-point rating scale (ranging from 1 “not at all characteristic” to 7 “completely characteristic”) into a more convenient 5-point scale (ranging from 1 “strongly disagree” to 5 “strongly agree”). The change of rating phrase was done since we intended to measure a future expectation rather than a present state. The resulting ten items are shown in Table 1.

Next to the E-SuPer measure, several other relevant measures were included in this study for validation purposes. In both samples, employee’s regular work performance was measured with the Individual Work Performance Questionnaire (IWPQ) developed by Koopmans et al. (2013). Two subscales of the IWPQ were included for the purpose of this study, namely *Task Performance* (TP) (five items, e.g., “I managed to plan my work so that it was done on time”; Internal consistency, expressed by Cronbach’s alpha, is 0.84 in both samples) and *Counterproductive Work Behavior* (CWB) (five items, e.g., ‘I complained about unimportant matters at work’; Cronbach’s alpha is 0.84 in Sample A and 0.83 in Sample B). Participants were asked to rate the ten statements on a 5-point Likert scale ranging from (1) ’seldom’ to (5) ’always’.

*Employee vitality* is defined by the presence of positive well-being, energy, fitness, and/or aliveness [39,40,41,42]. This refers to the extent to which an employee feels vigorous, as opposed to having a negative focus on the feeling of being fatigued and exhausted [17]. Therefore, employee vitality was measured using the Shirom–Melamed Vigor Measure [41], which is a 12-item instrument with three subscales. The items were rated on a seven-point Likert scale from (1) ‘never or almost never’ to (7) ‘always or almost always’. The SMVM assesses three dimensions of vigor; that is, physical strength (PS), cognitive liveliness (CL), and emotional energy (EE). Physical strength consists of five items; a sample item is “I feel I have physical strength”. Cronbach’s alpha was 0.93 for Sample A and 0.88 for Sample B). Three items represent cognitive liveliness; a sample item is “I feel I can think rapidly”. The internal consistency was 0.86 for Sample A and 0.88 for Sample B. Finally, four items were used for emotional energy; a typical item is “I feel able to be sensitive to the needs of coworkers and customers. Cronbach’s alpha is 0.94 for Sample A and 0.92 for Sample B.

### 2.3. Statistical Analysis

To examine the robustness of the psychometric analyses, a cross-validation procedure was followed [43]. In line with this procedure, Sample A was used as a calibration sample, and Sample B as a validation sample.

The validity of the measure of E-SuPer was examined in both samples, according to the definition and types of validity provided. The main focus in the present study is on factorial validity, being a rigorous test of construct validity [44]. We conducted exploratory factor analysis (EFA) using SPSS 26.0 (SPSS Inc., Chicago, IL, USA) for Sample A (calibration sample). More specifically, principal axis factoring (PAF) extraction with oblimin rotation where applicable was performed. The Kaiser-Meyer-Olkin (KMO) measure was used to quantify whether the items correlated sufficiently in order to determine whether a factor analysis could be performed. The decision regarding the number of factors to be retained was based on the Guttman–Kaiser eigenvalue greater-than-one rule. Meyers et al. (2013) [45] indicate that a guide for variance accounted for by the factors needs to meet the lower limit of 50%.

For validation Sample B, a confirmatory factor analysis (CFA) was conducted using RStudio Version 4.0.3. Next to the overall statistical fit index (chi-square—χ^2^), several practical fit indices were used, such as the Non-Normed Fit Index (NNFI), comparative fit index (CFI), the root mean square error of approximation (RMSEA), and the ratio of chi-square and degrees of freedom (χ^2^/*df*). According to Hair et al. (2014) [44] the values for these indices should exceed.90 for CFI and NNFI, RMSEA should be less than or equal to 0.08, and χ^2^/*df* should be lower than 5.0. If necessary, modification indices were used to improve model(s) by identifying parameters that, if included or deleted, would improve model fit. To assess the internal consistency of the measures, Cronbach’s alpha coefficient was determined, with 0.70 or higher being an acceptable value [46].

Finally, both concurrent and discriminant validity were assessed, which refer to the ability of a scale to behave as expected with respect to some other constructs to which it is related [47]. More specifically, we used the average variance extracted (AVE) test [48] for discriminant validity. Concurrent validity was demonstrated using Pearson zero-order correlations between the concerned measures.

## 3. Results

### 3.1. Initial Validity of the E-SuPer Instrument

First, an exploratory factor analysis and a confirmatory factor analysis were performed in Sample A and Sample B, respectively.

### 3.2. Exploratory Factor Analysis (Sample A)

The factor structure of the ten items of the E-SuPer instrument was investigated using PAF with oblimin rotation on Sample A (*n* = 153). Both the Bartlett test of sphericity (χ^2^ = 1126.27, *p* < 0.001) and the KMO test (0.90) indicated that the correlation matrix was factorable (>0.60). Based on the extraction of eigenvalues greater than 1, one factor was extracted, explaining 58.10% of the variance. Factor loadings ranged from 0.60 to 0.85 (*M* = 0.76), and all communalities were substantially higher than 0.30 (*M* = 0.58). See Table 1 for further details.

### 3.3. Confirmatory Factor Analysis (Sample B)

EFA suggested that our E-SuPer instrument can best be considered as a single-factor construct. To validate the single-factor structure, we performed a CFA in Sample B. The initial model (M_0_) was a one-factor model with all ten items loading on this factor and uncorrelated unique terms.

Table 2 shows that model M_0_ initially did not fit the data very well, χ^2^ = 90.07; *df* = 35, *p* < 0.001; χ^2^/*df* = 2.57; RMSEA = 0.10 (90% CI = 0.07–0.13); NNFI = 0.88; CFI = 0.91; AGFI = 0.84. Looking at the modification indices showed an improved model fit in relaxing the relation between item 2 and item 3. The modified factor model (M_1_, one-factor with one additional correlation) showed a better model fit than M_0_ (Δχ^2^ = 12.72, Δ*df* = 1, *p* < 0.001), but still did not sufficiently meet all thresholds: χ^2^ = 77.35, *df* = 34, *p* < 0.001; χ^2^/*df* = 2.28; RMSEA = 0.09 (90% CI = 0.06–0.12); NNFI = 0.90; CFI = 0.93; AGFI = 0.86.

A second look at the modification indices of model M_1_ showed a better model fit in case of freeing the relation between item 6 and item 7 as well (Table 2). The corresponding model M_2_ (one-factor and two additional correlations) showed a better fit than M_1_ (Δχ^2^ = 9.99, Δ*df* = 1, *p* < 0.001), and fitted the data well: χ^2^ = 67.36, *df* = 33, *p* < 0.001; χ^2^/*df* = 2.04; RMSEA = 0.08 (90% CI = 0.05–0.11); NNFI = 0.92; CFI = 0.94; AGFI = 0.88.

**Figure 2:**
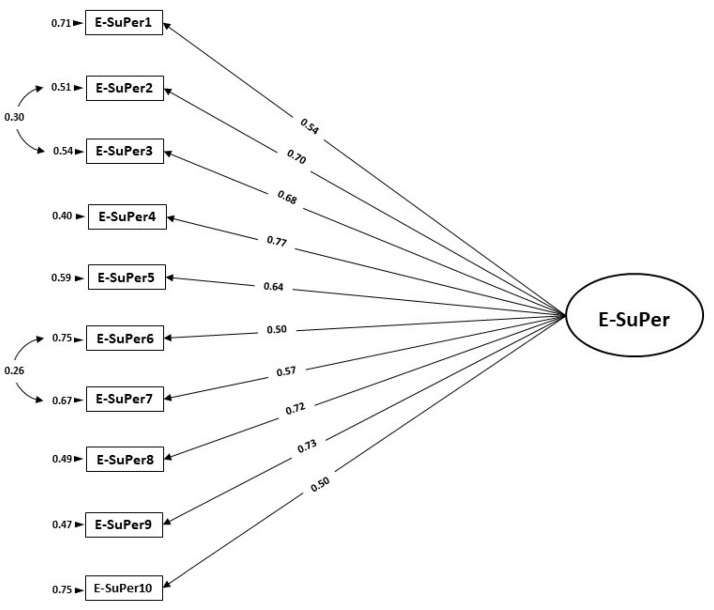
Final CFA model M_2_ and standardized factor loadings for the E-SuPer instrument (Sample B; *n* = 160).

As no further significant modification indices were detected, we accepted model M_2_ as a reasonable approximation of the underlying factor structure (see Table 2). Figure 2 represents the parameter estimates for model M_2_ of the E-SuPer instrument. The standardized factor loadings of the ten items ranged from 0.50 to 0.77 (*p* < 0.05).

### 3.4. Internal Consistency of the E-SuPer Instrument

The internal consistency of the E-SuPer instrument, expressed in Cronbach’s alpha, achieved 0.93 in Sample A, and 0.87 in Sample B. All item-total correlations were 0.66 or higher in both samples. There was no need for skipping items based on a substantial increase in alpha according to the Spearman–Brown prediction formula [49].

### 3.5. Construct Validity of E-SuPer with Closely Related Constructs

To further check the construct validity of the E-SuPer instrument, we employed both an EFA (Sample A) and a CFA (Sample B) on E-SuPer and its closely related constructs. Specifically, (1) E-SuPer with Task Performance (TP) and Counterproductive Work Behavior (CWB), and (2) E-SuPer with Vigor by means of its underlying subscales (PS, CL, EE).

### 3.6. Exploratory Factor Analysis (Sample A)

First, an EFA was applied for E-SuPer with TP and CWB, which was based on PAF extraction Oblimin rotation on Sample A. For this factor structure, three factors were found explaining 57.60% of the variance. The size of the KMO was 0.90, and Bartlett’s test of sphericity was χ^2^ = 1975.46, *p* < 0.001, indicating that all items had an adequate common variance for EFA. The three factors found adequately represented the 20 items in the expected way (factor loadings > 0.50). Only two items from E-SuPer instrument loaded on another dimension (task performance); that is, items 8 and 9.

The EFA for E-SuPer with the three vigor subscales PS, CL, and EE structure revealed four factors explaining 68.96% of the variance. The size of the KMO was 0.90 and Bartlett’s test of sphericity was χ^2^ = 2801.19, *p* < 0.001, indicating that the 22 items had an adequate common variance for EFA. The four factors detected showed an adequate representation of 21 items (factor loadings > 0.50). Only one item (item 6 from CL) showed an inadequate factor loading and overlapped between the factors PS (0.34) and CL (0.32).

### 3.7. Confirmatory Factor Analysis (Sample B)

**Figure 3:**
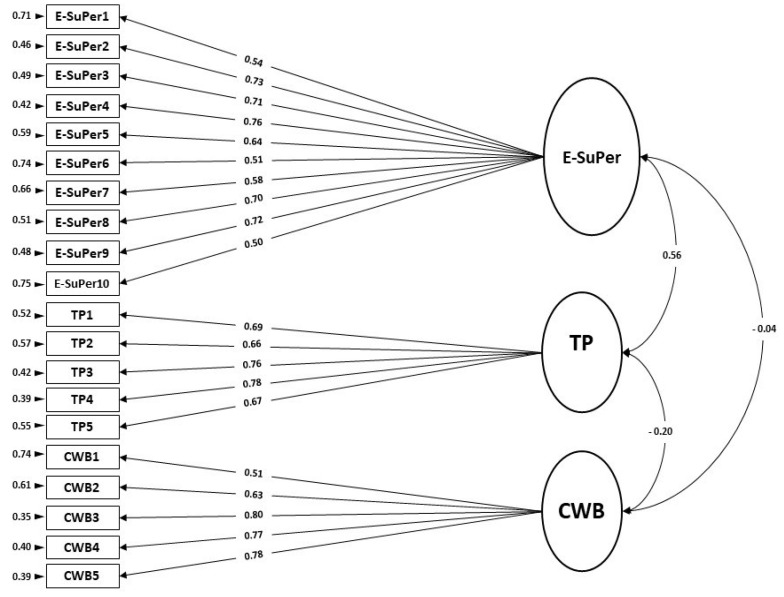
Path diagram and the coefficient for the three-factor CFA structure of E-SuPer–TP–CWB (*n* = 160).

Using CFA for validation purposes, we tested a three-factor model for E-SuPer with Task Performance (TP) and Counterproductive Work Behavior (CWB). A three-factor model where the items of SuPer-TP-CWB load on their respective factors was analyzed (see Figure 3). The fit indices values were found to be χ^2^ = 254.47, *df* = 167, *p* < 0.001; χ^2^/*df* = 1.52; RMSEA = 0.06 (90% CI = 0.04–0.07); NNFI = 0.92; CFI = 0.93; AGFI = 0.83. This model showed good fit indices values in each indicator, and modification indices were not necessary to apply in this model.

**Figure 4:**
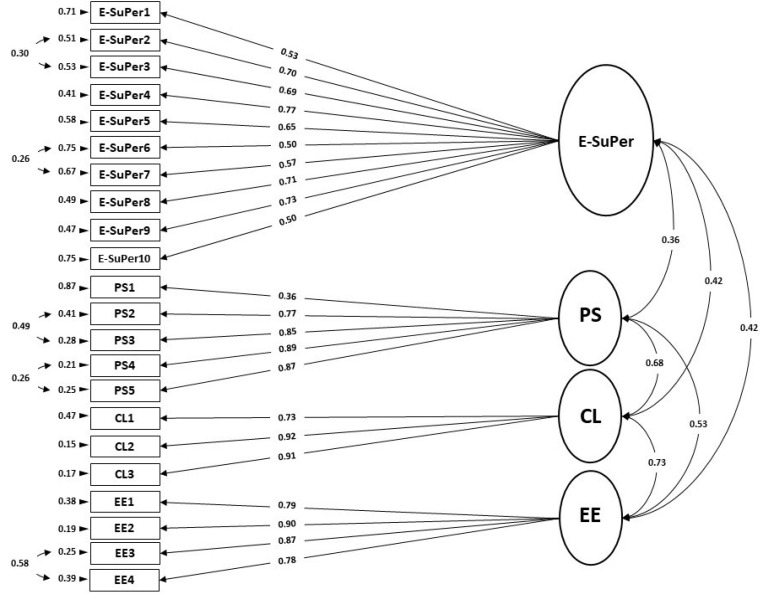
Path diagram and the coefficient for the four-factor CFA structure of E-SuPer–PS–CL–EE (*n* = 160).

As far as E-SuPer and the vigor subscales are concerned, we tested a four-factor model where the items of E-SuPer, PS, CL, and EE load on their respective latent factors. The fit indices values were found to be χ^2^ = 457.44, *df* = 203, *p* < 0.001; χ^2^/*df* = 2.25; RMSEA = 0.09 (90% CI = 0.08–0.10); NNFI = 0.87; CFI = 0.89; AGFI = 0.75. After revised by several suggested modification indices between within-factor error terms (see Figure 4), the fit indices for this four-factor model were acceptable: χ^2^ = 365.09, *df* = 198, *p* < 0.001; χ^2^/*df* = 1.84; RMSEA = 0.07 (90% CI = 0.06–0.08); NNFI = 0.91; CFI = 0.93; AGFI = 0.80. Therefore, the unidimensional structure of E-SuPer was further confirmed by this construct validation.

### 3.8. Discriminant and Concurrent Validity of the E-SuPer Instrument

To further validate the E-SuPer instrument, we examined the discriminant and concurrent validity of E-SuPer in relation to TP, CWB, PS, CL, and EE. To assess discriminant validity, the value of average variance extracted (AVE) square root for all constructs should exceed the squared inter-construct correlation in all the cases [47]. Findings in Table 3 provide empirical support for discriminant validity in that the AVE square roots of the six constructs were in the range of 0.72–0.90, which is larger than the correlation coefficient for each pair of constructs.

To check concurrent validity, Table 3 reveals that E-SuPer was significantly positively related to TP (Sample A: *r* = 0.67, *p* < 0.01; Sample B: *r* = 0.49, *p* < 0.01), and significantly negatively related to CWB in Sample A (*r* = −0.28, *p* < 0.01) but not in Sample B (*r* = −0.05, *p* = n.s.). Furthermore, E-SuPer was significantly positively related to PS (*r* = 0.32 in both samples, *p* < 0.01) and CL (Sample A: *r* = 0.43, *p* < 0.01; Sample B: *r* = 0.34, *p* < 0.01). Finally, E-SuPer was significantly positively related to EE (Sample A: *r* = 0.35, *p* < 0.01; Sample B: *r* = 0.38, *p* < 0.01).

## 4. Discussion

The aim of this paper was (1) to introduce a working definition of employee sustainable performance, and (2) to launch a corresponding instrument. With respect to the latter aim, a sub-purpose was to psychometrically test the instrument using two cross-sectional survey studies (Sample A, *n* = 153, and Sample B, *n* = 160) with a cross-validation procedure. Specifically, we focused on construct (i.e., factorial) validity, reliability (i.e., internal consistency), discriminant and concurrent validity.

Regarding the first aim, this study introduced a novel concept called employee sustainable performance (abbreviated E-SuPer), which was defined as a regulatory process in which an individual worker enduringly and efficiently achieves particular desired work goals while maintaining satisfactory levels of well-being. The three central parts in this definition focus on (a) the *balance* between employee performance (individual workers must efficiently achieve particular desired work goals) and employee well-being (that should be satisfactory); (b) the long-term aspect of this balance (workers must enduringly achieve a particular level of performance, while well-being must be maintained; and (c) a regulatory process that leads to an acceptable, long-term balance between employee performance and well-being (i.e., to regulate behavior, to guide thoughts, feelings, and actions over time, as well as changing circumstances to attain goals [50,51].

Based on this working definition, we developed a ten-item measure that was based on existing measures of performance, focusing on workers’ expectation to be able to maintain a particular level of performance in the future, called the E-SuPer instrument. This measure was subsequently cross-validated in two independent samples. As far as construct validity by means of factor analysis is concerned, our findings indicated that the E-SuPer instrument has a good single-factor structure on Sample A by EFA, which was successfully validated in Sample B by CFA, though with minor modifications in two error terms relations. The reliability of the E-SuPer instrument in the calibration sample also emerged in the validation sample, demonstrating good internal consistency across both samples [46]. Hence, it is reasonable to conclude that the proposed measure is internally consistent.

The results of discriminant validity further suggest that the construct of employee sustainable performance, as measured by the E-SuPer instrument, is a unique construct and discriminates from other relevant constructs, such as task performance, counterproductive work behavior, or employee vitality. As for the concurrent validity, the relations between E-SuPer and other constructs were largely as expected. Highly employee sustainable performance was in general positively associated with indicators of task performance, physical strength, cognitive liveliness, emotional exhaustion (Hypotheses 1–2 supported) and negatively with counterproductive work behavior in Sample A (Hypothesis 3 supported). The lack of association between E-SuPer and counterproductive work behavior in Sample B (Chinese employees) could be due to cultural issues. From the perspective of divergent theorists, people in different social and cultural contexts might be shaped different conceptions about what behaviors constitute counterproductive work behavior. In other words, social and cultural context may impact, to a greater or lesser extent, the specific content of counterproductive work behavior in relation to employee sustainable performance [52]. On the other hand, it could be that performing well on one’s core tasks that also abstains from counterproductive work behavior is more prominent in Western countries [53].

Finally, our cross-validation method showed rather robust findings between the two samples. All these results indicated promising preliminary psychometric properties of the E-SuPer instrument to measure employee sustainable performance.

### 4.1. Theoretical Implications

The current paper has several theoretical implications. This study brings the notion of E-SuPer out of its academic black box, and provides a very first working definition as well as a new instrument. Employee sustainable performance takes root and evolves from considerations of previous research on concepts that focus on work performance and employee well-being from both a short-term as well as a long-term career perspective.

Our results are in line with the view of De Jonge and Peeters [1] and Dorenbosch [17], who have concluded that employee vitality is important for employee sustainable performance. Findings are also in agreement with the work of Porath and her team [14], who theorized about thriving at work (i.e., active learning and vitality) as crucial for employee sustainable performance at work.

Employee sustainable performance is associated with both high levels of employee performance and high (at least satisfactory) levels of employee well-being [1]. It is, therefore, broadening the scope of emphasizing the longer-term sustainability of high employee performance, which would be beneficial to both the employee and the organization [13,15]. On the other hand, our research has not only developed a measure that can be used to examine E-SuPer empirically, but also provide novel insights in this matter, opening up new avenues for research in the area of human resources management as well as work and organizational psychology. The ultimate goal is to build a better theoretical framework and to promote the progress of the entire related academic field. Thus, the construct of E-SuPer makes a unique theoretical contribution to the explanation of closely related variables, and the new definition of E-SuPer and its operationalization has been proved by the present study.

### 4.2. Strengths and Limitations

This study has several strengths and limitations. An obvious strength was that we developed the E-SuPer measurement instrument and conducted a very first empirical study on employee sustainable performance, which has not been done before. Moreover, our instrument was developed and tested using two different samples of survey data, which allowed for cross-validation of findings. Although this is a strength of the present study, it should be noted that one of these samples (Sample A) consisted of diverse employees working in a range of organizations across the world (but came mostly from Europe), whereas the other sample (Sample B) included participants from China. This limitation implies that one needs to be cautious in generalizing these findings to other countries or cultures, since data from other countries or cultures besides Europe and China are lacking.

The second limitation would be underlying comprehension bias and contextual differences. For reasons of cross-validation, the questionnaires of this study were conducted in two language versions (i.e., English and Chinese). Although the translation and back-translation procedures were strictly followed, comprehension bias due to the different cultures and contexts could not be ruled out. However, our psychometric findings did not show convincing evidence that our results were heavily biased by different cultural and contextual factors. In addition, a comparison of the two samples indicated that Sample A showed a significantly higher educational level and work experience than Sample B. From the perspective of affecting employee performance and well-being, these two demographics might have impacted the responses and generalizability of the present study. However, Pearson zero-order correlations between these variables showed small to modest associations in both samples, indicating no high impact of sample differences in education and work experience on our outcomes.

Furthermore, it is worth noting that the COVID-19 pandemic as a global health crisis has affected most employees’ working behavior and well-being experience, and even their career life [54]. Our samples were subjective reflections of the psychological states of employees both during and after this pandemic. For example, in Sample A, 91.5% of the participants reported that their countries were facing COVID-19 lockdown regulations, and 78.4% of participants were working from home. However, Sample B was collected in China, which had been realized in the post-pandemic era in which employees had basically returned to normal work and life. Therefore, despite cross-validation of our survey data, further generalization of the E-Super instrument is recommended. A third limitation is that our study had a cross-sectional research design, which means it did not cover large or multiple time intervals which are needed to investigate employee sustainable performance over time. Although cross-sectional research is necessary and pivotal in exploratory and replication research, longitudinal research methods could investigate these kinds of constructs more profoundly [55].

Fourth, the nature of the E-SuPer construct might reflect a possible overlap extent with other similar constructs, such as task performance, which might lead to confusion in the measurement results of these constructs However, conceptual discussions and methodological results in this study might help to differentiate these constructs to some extent.

Fifth, further validation should be based on additional sets of variables. Although we depicted relations of tests with related variables which have been established in earlier research (e.g., employee task performance; employee vitality, counterproductive work behavior), validity was examined using a limited set of variables. Preliminary findings were promising, but further validation research would seem desirable, for instance with respect to possible antecedents (e.g., job demands and job resources) and other kinds of outcomes (e.g., employee health). In this respect, research on the role of self-regulation and longer-term outcomes is needed as well to test the presumed beneficial effects of E-SuPer.

A final limitation is that the initial confirmatory factor analysis on Sample B did not fit the model very well. Therefore, the modification indices (MIs) were detected in identifying sources of misfit and applied to modify the single factor construct to be fitted. However, using MIs also carries risks. First, they are merely determined by Sample B data and not based on theory. Second, simulation research has suggested that using MIs to guide model specification rarely leads to the true population model [56]. Thus, the E-SuPer instrument should be further validated in other relatively large, diverse and well-collected samples.

### 4.3. Future Research

The first avenue for future research could be to check the psychometric analyses of the E-Super instrument in other more representative geographical locations and cultures. Second, future studies could use longitudinal designs, covering large and multiple time intervals, to test further predictive validity for clarifying the relative uniqueness of the E-SuPer instrument. A third route for future research could be based on additional sets of variables. Many related antecedents and consequences of E-SuPer have been proposed and assumed over the last decade, indicating that the effectiveness of E-SuPer can take multiple forms. To provide a more holistic picture, future validation may include: possible antecedents (e.g., job demands), moderators (e.g., job resources, recovery, personal characteristics, self-regulation), and an enlarged set of outcome criteria (e.g., job satisfaction, organizational commitment, work engagement, burnout, health complaints) with data gained from multiple sources (e.g., supervisor ratings of subordinate, objective measures of team/individual performance). We tend to believe that these additional variables will further strengthen the arguments supporting the predictive value of the E-SuPer instrument [1]. Finally, our research has mainly focused on (self-regulatory) processes occurring at work. Experiences and events happening in other achievement domains (i.e., sports) may also be related to sustainable performance. Gaining knowledge about how to enduringly and efficiently maintain performance in a different career, as well as life stages, are highly relevant for (elite) athletes as well. For instance, research on grit has shown that a person’s (usually refers to athlete) dispositional tendency towards passion and perseverance for long-term goals, which also positively impact goal-attainment and long-term success [57,58].

### 4.4. Practical Implications

A high level of work performance is crucial for both the employee and the organization—not only in the short term, but also and particularly in the long run. It should be noted, however, that E-SuPer implies that high employee work performance should not be achieved at the cost of employee well-being. Instead, high employee well-being may be considered a precondition for high work performance, as stated in the ‘happy-productive worker hypothesis’ [59,60]. Similarly, unhealthy workers are unlikely to perform well, and at the same time to be ‘sustainable’ at work. From an organizational perspective, organizations need employees to be able to be ‘equipped’ for sustainable work performance, to improve the competitiveness of the whole system in a healthy and satisfying manner. Ideally, this is an employee self-regulatory process that involves a continuous (re)generation and preservation of resources invested while achieving work performance goals. Since organizations must deal with demographic trends such as aging, longer working lives, and stable youth growth, keeping employees working and productive for a “lifetime” while being vital and healthy is necessary and appealing. Therefore, we argue that exploring employee sustainable performance and promoting its formation is of great significance to employee well-being and development, to the organizations these employees work for, as well as meaningful for building social sustainability.

Furthermore, the E-SuPer instrument developed in this study may also serve as an empirical vehicle for getting a better understanding of the degree to employee work performance as well as anticipating their well-being level. Implementing such practices of E-SuPer has been noted by scholars to be an important aspect for creating mutual interests between employees and their organizations that could aid in the reduction of negative aspects in the workplace, such as employee resignation, doctor visits, burnout, and work-home conflict [1,13,14]. By linking such features to individual attitudes and working behaviors, this tool may also be useful for assessing and improving the effectiveness of E-SuPer initiatives. Furthermore, by linking such features to individual-level outcomes (e.g., job satisfaction, work engagement, organizational commitment, organizational citizenship behavior), this tool may be useful for conducting comparisons on the relations between various approaches to individual work performance and employee well-being.

## 5. Conclusions

Building on recent insights in both the performance and sustainability literature, the key contributions of this study are its preliminary evidence for a conceptualization of employee sustainable performance. In addition, it develops and cross-validates the first measure of this concept, the so-called E-SuPer instrument. Specifically, empirical evidence was found for its validity and reliability, and the psychometric properties of the E-SuPer scale appear to be satisfactory across two samples. Further, research using the E-SuPer construct may help researchers and practitioners to gain a thorough understanding of the job-related factors associated with high employee performance and well-being in the long run. Finally, the E-SuPer instrument has the potential to be conceptualized as a positive psychological state of employees at work. Future research may provide further evidence for the usefulness of this novel concept for both theory and practice.

## Figures and Tables

**Table 1:** Items, means, standard deviations and factor loadings of the E-SuPer instrument (Sample A, *n* = 153).

*During My Entire Career, I Will Be Able to …*		*M*	*SD*	Factor Loading (Communality)
1	… continuously achieve the objectives of my job.	3.88	0.83	0.78 (0.61)
2	… permanently meet the criteria for my job performance.	3.84	0.90	0.85 (0.72)
3	… continuously demonstrate expertise in all job-related tasks.	3.91	0.88	0.75 (0.56)
4	… persistently perform well in the overall job by carrying out tasks as expected.	4.00	0.73	0.84 (0.70)
5	… continuously fulfill all the requirements of my job.	3.98	0.82	0.85 (0.72)
6	… permanently be competent in all areas of my job.	3.71	0.93	0.82 (0.67)
7	… persistently manage more responsibility than typically assigned.	3.74	0.92	0.60 (0.36)
8	… organize and plan well to achieve objectives of my work in a sustainable way.	3.69	0.95	0.71 (0.51)
9	… organize and plan well to meet deadlines of my work in a sustainable way.	3.69	0.96	0.68 (0.46)
10	… permanently be suitable for my job.	3.99	0.80	0.71 (0.51)

**Table 2:** Fit Values of the E-SuPer Scale for Sample B (*n* = 160).

	Description	χ^2^/*df*	RMSEA	NNFI	CFI	AGFI
Model_0_ (M_0_)	One-factor model	2.57	0.10	0.88	0.91	0.84
Model_1_ (M_1_)	M_0_ + error terms of items 2–3 correlated	2.28	0.09	0.90	0.93	0.86
Model_2_ (M_2_)	M_1_ + error terms of items 6–7 correlated	2.04	0.08	0.92	0.94	0.88

**Table 3:** Descriptive statistics and zero-order Pearson correlations for Sample A (below the diagonal, *n* = 153) and for Sample B (above the diagonal, *n* = 160).

	Sample A	Sample B						
	*M*(*SD*)	*M*(*SD*)	1	2	3	4	5	6
1. E-SuPer	3.84 (0.68)	3.50 (0.51)	**0.75 (0.64)**	0.49 **	−0.05	0.32 **	0.34 **	0.38 **
2. TP	4.34 (0.69)	3.49 (0.64)	0.67 **	**0.72 (0.72)**	−0.16 *	0.43 **	0.48 **	0.49 **
3. CWB	3.84 (0.79)	2.62 (0.69)	−0.28 **	−0.44 **	**0.72 (0.73)**	−0.08	−0.01	−0.12
4. PS	4.35 (1.16)	4.16 (1.03)	0.32 **	0.46 **	−0.42 **	**0.85 (0.80)**	0.64 **	0.46 **
5. CL	3.84 (1.18)	4.19 (1.12)	0.43 **	0.53 **	−0.27 **	0.64 **	**0.84 (0.86)**	0.65 **
6. EE	4.35 (1.28)	4.50 (1.15)	0.35 **	0.39 **	−0.23 **	0.53 **	0.59 **	**0.90 (0.86)**

Note: Bold figures on the diagonal are the square root of AVE figures for Sample A (outside brackets) and Sample B (inside brackets). * *p* < 0.05 level (2-tailed), ** *p* < 0.01 level (2-tailed).

## Data Availability

Data presented in this study are available upon request from the corresponding author.
